# Enhancing biology and providing structural support for acetabular reconstruction in single-stage revision for infection

**DOI:** 10.1186/s10195-019-0530-6

**Published:** 2019-06-24

**Authors:** Ayman M. Ebied, Ahmed A. Ebied, Sameh Marei, Evert Smith

**Affiliations:** 1 Orthopaedic Department, Faculty of Medicine, Menoufia University Hospitals, Gamal Abdel Nasser Street, Shebin El kom, Menoufia Governorate, Egypt; 2 Southmead Hospital, Westbury on Trym, Bristol, UK

**Keywords:** Tantalum augments, Antibiotic-loaded impaction graft, Single-stage revision, Periprosthetic infection, Cemented cup

## Abstract

**Background:**

Reconstruction of combined segmental and cavitary defects of the acetabulum is a challenge to the hip surgeon. One question regards the efficacy of reconstruction of acetabular defects using a combination of tantalum metal augments (TMAs) and impaction graft in single-stage revision for periprosthetic infection.

**Materials and methods:**

In the period between July 2009 and August 2014, 24 patients with combined segmental and cavitary acetabular defects and Paprosky classification grade IIB, IIC, and IIIA had hips reconstructed using the combination of TMAs and antibiotic-loaded impaction grafting. A similar group of 30 patients who received single-stage revision without metal augments were identified and taken as control. All patients received a polyethylene cemented cup and long cementless (Wagner SL) stem. Patients were prospectively evaluated using the modified Harris Hip Score (HHS) in addition to radiological evaluation at 3, 6, and 12 months then annually thereafter.

**Results:**

At an average follow-up period of 4 years (range 2–7 years), all but one patient in the study group were free of infection, indicating a 96% success rate. This rate of eradicating infection was comparable to the 97% success rate in the control group. All metal augments were stable, and good incorporation of the impacted bone graft was observed. The HHS improved significantly from 27 preoperatively to 83 postoperatively (*P* < 0.001).

**Conclusion:**

Metal augments can convert massive acetabular defects to a more contained defect suitable for grafting. The combination of tantalum augments that provide strong structural support and antibiotic-loaded allograft is successful in the mid-term in single-stage revisions for infection.

**Level of evidence:**

Level IV (prospective case series).

## Introduction

Acetabular defects are commonly seen in revision hip arthroplasty [22]. Various strategies have been suggested to overcome acetabular bone deficiencies, including bone graft (bulk or morselized), metal mesh, cages of various designs [12], and finally tantalum metal augments [7, 26].

The advantages of using antibiotic-loaded impaction graft in acetabular defects are restoring the bone stock [24], enhancing biologic reconstruction, and delivering high doses of antibiotics needed in cases of infection [4, 9, 30].

However, impaction bone grafting is technically demanding, especially in cases of combined segmental and cavitary defects [3, 15]. When segmental defects are faced, addition of metal meshes becomes necessary to convert the defect into a contained state and/or use of metal cages to protect the impacted cancellous chips.

The use of metal cages is fraught with difficulties and potential loosening at a later stage. Med-term outcomes using cages for acetabular defects show a considerable rate of loosening and failure of the construct [14].

Tantalum metal augments have a highly porous surface with early bone and soft tissue in- and on-growth [10]. Such augments have been successfully used to overcome segmental acetabular defects, even in revision for infection [17]. Additionally, the porous tantalum material showed a lower incidence of infection in cementless revision total hip replacement (THR) [27]. Hence, metal augments provide a stable and durable construct when reconstructing acetabular defects.

Could the combination of bone graft and metal augments be successfully used, and what are the advantages of this combination? This article presents the technical details and outcome of this approach for acetabular reconstruction in single-stage revision for periprosthetic hip infection.

## Materials and methods

This is a prospective study of 74 patients with periprosthetic infection of the hip, who received single-stage exchange arthroplasty in the period between July 2009 and August 2014. Inclusion criteria for performing single-stage revision were absence of actively draining sinus tract and/or acute septicemia. Identification of the infecting organism by preoperative aspiration of the hip and availability of suitable antibiotic(s) according to culture and sensitivity tests was ensured in all patients. Twenty-four of the 74 patients had combined segmental and cavitary acetabular defects. These 24 patients had acetabular reconstruction using a combination of tantalum metal augments (TMAs) for acetabular wall reconstruction and impaction graft to the floor of the acetabulum using antibiotic-loaded cancellous allograft.

Data for the remaining patients were searched, and a similar group of 30 patients who had not received metal augments in their acetabular reconstruction were identified. These 30 patients were used as a control group.

McPherson’s grading scheme for local and systemic risk factors was utilized in the preoperative categorization of patients in this study cohort [20].

The posterior approach with additional extended trochanteric osteotomy (ETO) was employed in all patients. However, details of the approach were occasionally modified to permit debridement of infected tissues tracking outside the hip space.

Extensive debridement of all obstructive infected bony and soft tissue was performed to expose the hip. Removal of all implants and bone cement was performed. The addition of ETO allowed good visualization and excision of the hip capsule and infected membranes, particularly on the femoral canal and inner surface of the reflected lateral wall of the proximal femur. Irrigation with saline using pulsed lavage was done after debridement. The acetabulum and femur were then packed with H_2_O_2_-soaked gauze for 15–20 min while change of drapes, surgical gowns, and surgical instruments was performed, prior to the reconstruction and implantation phase. Infusion of intravenous antibiotic(s) was commenced after collection of a minimum of six specimens from the fluid and membranes at the implant bone interface. Standard and extended cultures (2 weeks) on blood agars were performed for the collected specimens, in addition to antibiotic sensitivity tests.

**Fig. 1:**
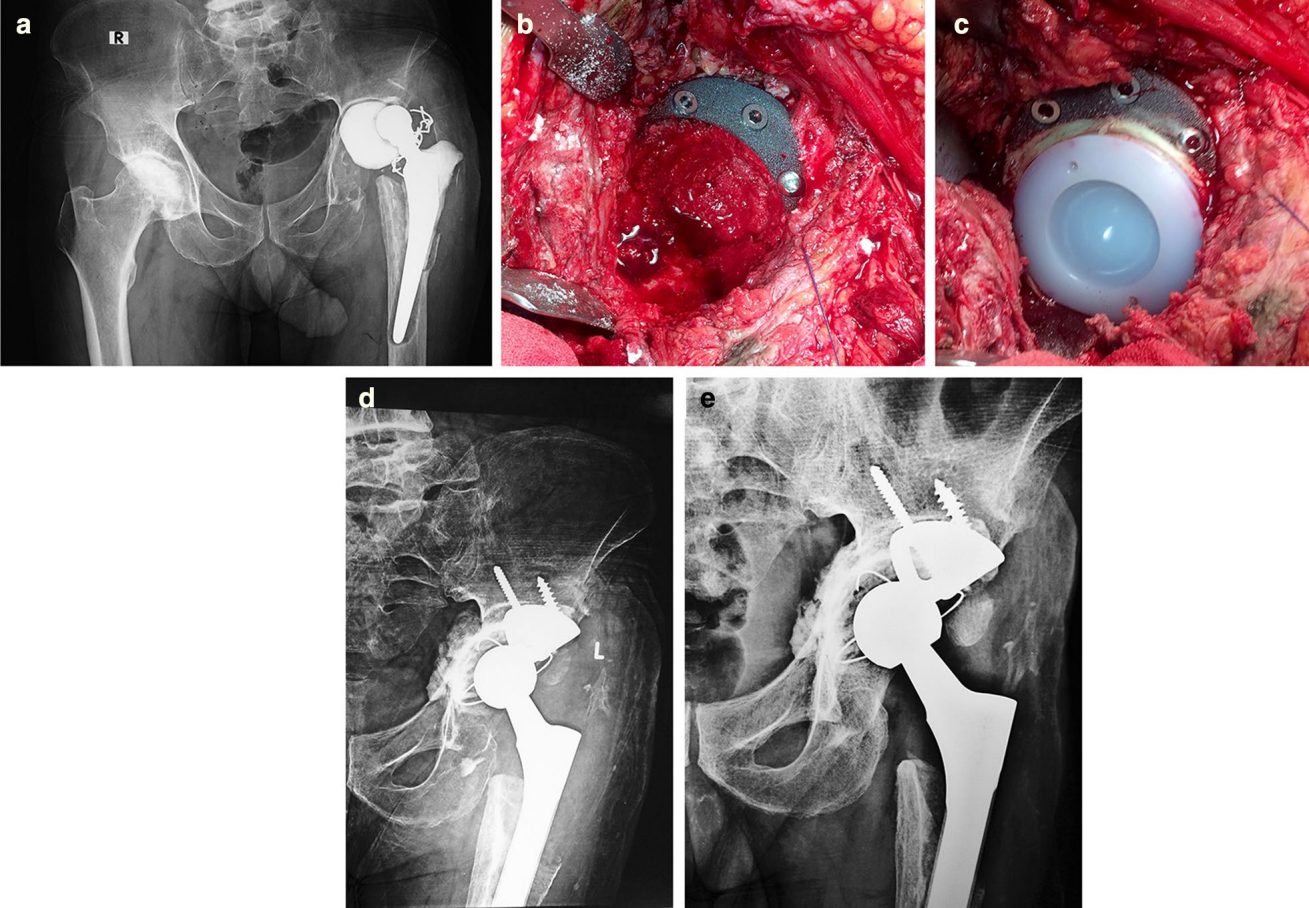
**a** Preoperative X-ray for a 62-year-old patient with infected hip spacer and acetabular defect grade IIIA. **b** Intraoperative photo showing insertion of 15-mm TMA that covers the superior acetabular defect. AB-loaded graft is impacted into the medial acetabular defect. **c** All-polyethylene cup is cemented into position and supported by the superior metal augment. **d** An X-ray 6 months postoperatively shows the cup position and bone graft. The cup is 5 mm above the hip anatomic center of rotation. **e** Follow up X-ray after 2 years shows maturation of the impaction graft, stable cup with no radiolucent lines, and well-integrated TMA

*Acetabular reconstruction:* following removal of the implants and debridement of infected membranes, the extent of acetabular bone defect was reassessed. Tantalum metal augment that fits the segmental defect at the superior or posterosuperior aspect of the acetabulum was selected and fixed to viable bone using two or three screws, depending on the size and width of the augment. Having fixed the metal augment in place, several layers of morselized cancellous chips were impacted into the acetabular floor and remaining wall defects (the technique is presented in the case scenario of Fig. 1a–e).

**Fig. 2:**
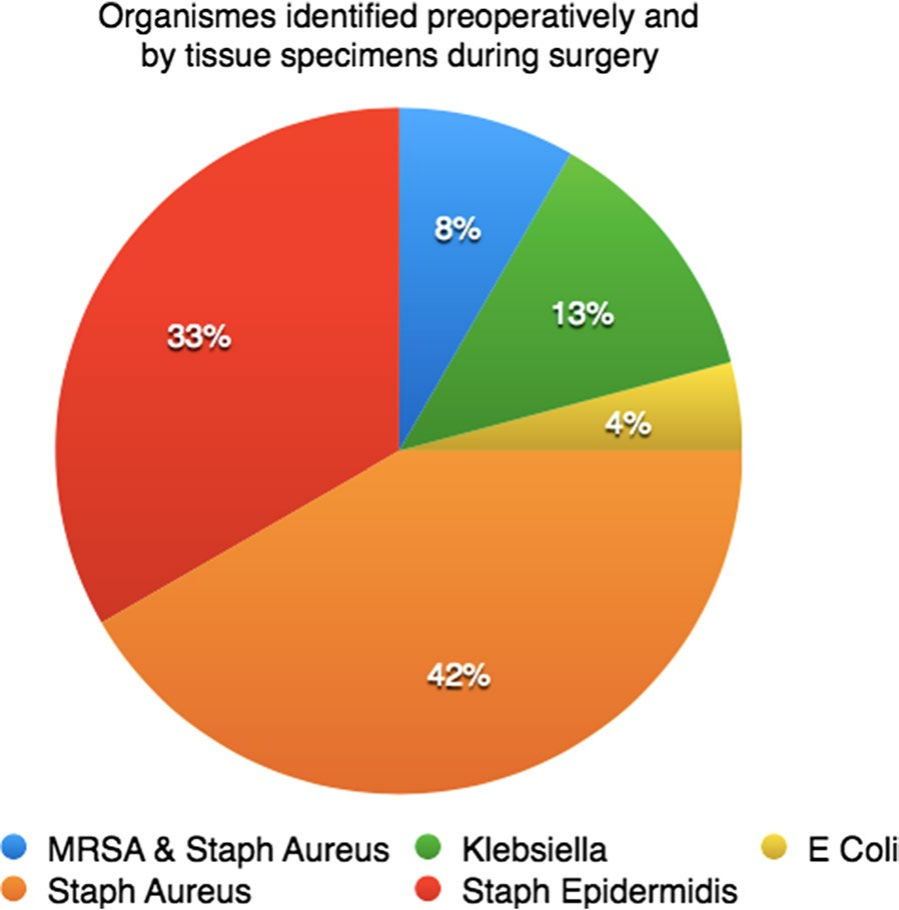
Graph showing organisms identified by preoperative aspiration and confirmed by intraoperative tissue specimens

Fresh, frozen femoral heads were sliced using power saw and rongeurs, and washed with saline, and 4 g of antibiotics in powder form was added per head. The choice of antibiotic was determined by culture and sensitivity tests performed preoperatively (Fig. 2). The usual combination was 2 g vancomycin and 2 g meropenem per head.

All-polyethylene cemented cups were inserted after pressurization of gentamicin-loaded bone cement (PALACOS R+G, Zimmer-Biomet). Thirty-two or 28 mm inner diameter high-cross-linked polyethylene cups were implanted (ZCA, longevity cross-linked all-polyethylene cup, Zimmer).

On the femoral side, Wagner SL straight flanged stems (Zimmer, Switzerland) were used in all patients. Reattachment of the trochanter was performed using stainless-steel double wires and Ethibond number 5 transosseous sutures for repair of the short external rotators.

Postoperatively, patients were given IV antibiotics for 6 weeks, and oral antibiotics (rifampin, linezolid, or fluoroquinolones) were continued for another 8 weeks. Touch weight bearing (WB) was allowed from the second postoperative day for 6 weeks. This was followed by partial WB for another 6 weeks before going to full WB at 12 weeks.

**Fig. 3:**
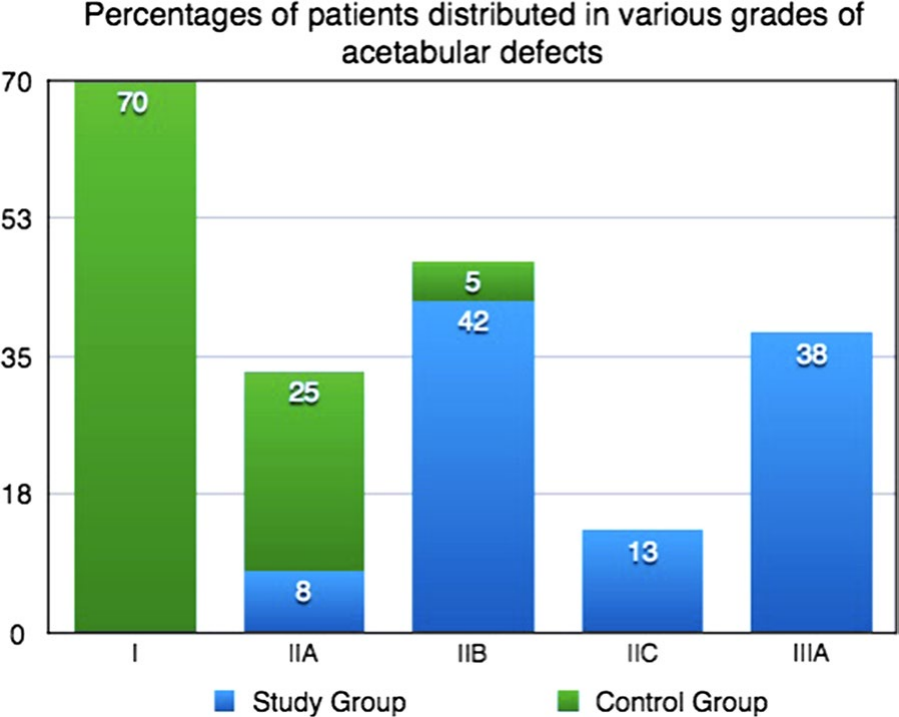
Incidence of various grades of acetabular defects in the study and control groups. Numbers on columns express the percentage among the overall number of patients in the specific group

The same management protocol was followed in the control group, apart from the use of metal augments for acetabular reconstruction. Acetabular defects were less severe and impaction bone graft was needed in only 10 patients (Fig. 3).

Radiological evaluation was performed at 3 and 12 months, and annually thereafter. Stability of the metal augments was assessed using four criteria: (a) change in position of the augment, presence of radiolucent line (b) at the augment–bone interface or (c) around the screws, and finally (d) breakage or backing out of the screws [1]. In an anteroposterior X-ray of the pelvis, inclination of the cemented cup was assessed in the horizontal and vertical planes. Any change in position of the cup by > 5° was considered to be evidence of loosening.

Any radiolucent lines in zones 2 and 3 according to Charnley and De Lee [8] were recorded. The impaction graft stability and incorporation were evaluated by the presence of crossing trabeculae between the host bone and the impacted graft [28].

Restoration of the hip center of rotation was assessed by measuring the distance in millimeters between the inferior edge of the cup and the inter-teardrop line on digitized and calibrated pelvic X-rays.

Hip function was prospectively evaluated using the Harris Hip Score (HHS) recorded preoperatively, at 12 months postoperatively, and annually thereafter.

Wilcoxon test was used to compare pre- with postoperative results as well as comparing the study with the control group; *P* < 0.05 was considered to indicate statistically significant difference.

## Results

Twenty-four patients formed the study group, with another 30 as control. These patients were followed for an average of 5 years (range 2–7 years). At the final evaluation, 2 patients from the study group and 4 from the control group had died from causes unrelated to the studied intervention, leaving 22 patients as the study group and 26 as controls.

**Fig. 4:**
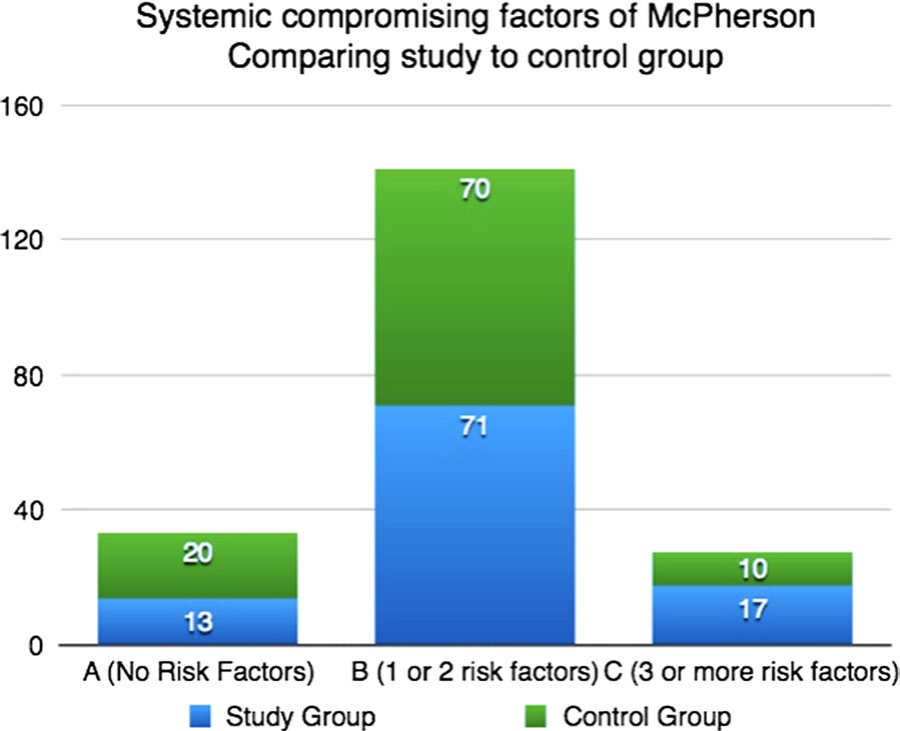
Percentage of patients in each of the three grades of McPherson classification for systemic compromising factors. Numbers on columns show percentages among the overall number of the specific group

The study group was comparable to the control group in terms of age, sex, and type of implants, as well as patients’ general and local risk factors according to McPherson’s classification (Fig. 4).

In the study group, 17/24 patients had late infection of their implants that appeared at an average of 5 years from primary arthroplasty (range 1–6 years). The other seven patients had early manifestations for infection within the first 4 weeks from the primary procedure. However, it took on average 12 months to receive their revision procedures.

According to McPherson’s classification, the study group had 3 patients (13%) who were grade A, while the majority (17/24, 71%) were grade B, with one or two systemic compromising factors. The remaining four patients (17%) had three or more systemic compromising factors and were classified as grade C. On the other hand, 17/24 (71%) had no local compromising factors with a grade I classification; 7/24 (29%) were grade II (Figs. 3, 4).

The most commonly encountered type of acetabular defect was grade IIB in 10/24 (42%). More severe defects of grade IIIA were seen in 9/24 (38%), while grade IIC and IIA were less commonly observed, in 13% and 8%, respectively.

Infecting organisms were identified in all patients. *Staphylococcus aureus* was the most common infecting organism. Other organisms were identified, but no fungal infection was discovered in this series (Fig. 2).

At an average follow-up of 5 years (range 2–7 years), all patients except one were free of infection, with 96% success rate. This success rate in eradication of infection was comparable to the 97% success rate of the control group, with one case of recurrence of infection.

Stability and integration of all TM augments was observed with no change in position or radiolucent lines recorded at the bone–augment interface or around any of the fixing screws.

The AB-loaded impacted cancellous grafts had been incorporated into the medial acetabular defects. No graft resorption was observed in all hips. Maturation of the graft with formation of bone trabeculae at the junction between the acetabular defect and host bone was observed in 20/24 hips (Fig. 1d, e).

Evaluation of bone–cement interface for the cemented cups was possible at Charnley and De Lee zones 2 and 3, as zone 1 of the cup was covered by the metal augment. None of the cemented cups was loose; however, nonprogressive radiolucent lines in zone 2 or 3 were observed in 5/24 cups.

Preoperative proximal migration of the hip center of rotation (HCR) from the anatomic position was 19 mm (range 10–35 mm). This elevation in HCR was significantly reduced to an average of 2 mm (range 0–10 mm) postoperatively (*P* < 0.001). The inferior edge of the cup was found at the level of the inter-teardrop line in 16/24.

Significant improvement of the patients’ hip function was recorded, with an increase in average HHS from 27 (range 24–31) preoperatively to 83 (range 75–90) postoperatively (*P* < 0.001).

Few postoperative complications were reported in this series. Serous discharge from their wounds for > 5 days postoperatively was recorded in 5/24 patients. Four of these five were hepatic patients and needed optimization of their serum albumin levels. However, one patient continued to have wound discharge and needed debridement of the wound 2 weeks after the revision surgery. The same patient had a recurrence of infection 6 months later and opted for a Girdlestone procedure to be performed.

## Discussion

Reconstruction of acetabular defects is a challenge that is commonly faced in revision for periprosthetic infection. Impaction graft has been reported to achieve high success rates in the long term [28]. Meanwhile, few articles have presented results of impaction graft in single-stage revision for hip periprosthetic infection [9, 24, 30]. However, the technique of morselized bone graft impaction showed less favorable outcomes when the defects were massive or needed the use of two metal meshes [3, 15]. The question that should then be asked is: how to obtain the benefits of using impaction graft that enhances the patient’s bone stock and can be loaded with the antibiotics necessary for the treatment of periprosthetic infection in massive combined segmental and cavitary acetabular defects?

TMAs have been successfully used to overcome segmental acetabular defects [7, 11]. Though trabecular metal cups were reported to have a low rate of infection in revision hip arthroplasty [27], the success of TMA in revision for infection has recently been reported from an arthroplasty institute [17]. It is important for the orthopedic community to discover whether similar results can be reproduced by different centers and health systems.

In this series of single-stage revisions for periprosthetic infection, TMAs that overcome segmental defects and provide structural support [10] were combined with antibiotic-loaded fresh frozen allograft that acts as an AB carrier [9, 31] and fills the contained medial acetabular defect [11]. High success rate in both eradicating infection and overcoming combined segmental and cavitary acetabular defects was achieved by employing this technique.

There are several advantages to using TMAs. First, they reduce the size of the acetabular defect necessary to be filled with bone graft. Additionally, they convert massive segmental defects that normally need the addition of metal meshes before grafting into contained ones, making impaction grafting less technically demanding. Finally, TMAs provide a stable construct for early weight-bearing mobilization of the patients.

It may be argued that metal cages can provide an alternative to the presented reconstruction technique. However, metal cages do not integrate with host bone and have considerable chance of loosening and early backing out [14]. Therefore, having a technique for reconstruction that easily incorporates into host bone may provide a better alternative. However, it remains to be seen whether this combination of TMAs and impaction graft will stand the test of time in the longer term.

Although two-stage revision for infection is considered the gold standard by many surgeons, there is some accumulating evidence that single-stage exchange for periprosthetic infection can achieve similar success rates when applied to suitable candidates [18]. Strict patient selection criteria with preoperative identification of the infecting organism, appropriate debridement of infected bone and soft tissues, and use of adequate doses of antibiotics both locally and systemically are key factors to achieving favorable outcomes [9, 18, 21].

Despite the fact that cementless implants are regularly used in two-stage revision protocols [19], some may question the safety of employing bone graft or cementless components in single-stage revision for infection. Earlier reports on single-stage revision were for cemented components on the femoral and acetabular sides [23]. However, as the technique became adopted by many European centers and is being used for patients with deficient bone stock, the value of bone graft and cementless implants cannot be ignored. High success rates ranging from 84% to 100% were reported with protocols of single-stage revision employing cementless implants on the acetabular and femoral sides [2, 30, 32]. Based on currently available evidence, both bone allograft and cementless components can be implanted in single-stage revision for infection with high rates of success. Cementless implants are less likely to adversely influence the outcome in single-stage revision protocols [2].

In this series, great attention was paid to the exposure of the acetabular and femoral sides of the hip. The posterior approach was employed in all cases in addition to an ETO. The reflection of the greater trochanter (GT) allowed removal of cement particles from the medullary canal as well as the infected membranes from the inner surface of the GT. On the acetabular side, the hip capsule was circumferentially excised before reaming the acetabular walls and starting the reconstruction. Occasionally, completion of the debridement required modification of the approach to reach other areas in the lateral compartment of the thigh that were invaded by infected tissues.

In this article, the study group of patients with massive acetabular defects was compared with a control group of patients who underwent a similar reconstruction technique. The only difference was in the extent of acetabular defects and the use of TMA for acetabular reconstruction. Similar rate of eradication of infection as well as cup stability and functional outcome was observed in the two groups. This indicates that the severity of the acetabular defect did not adversely affect the outcome when the combination of TMA and AB-loaded impaction bone graft was used for reconstruction.

Resistant bacteria such as methicillin-resistant *S. aureus* (MRSA) and Gram-negative bacteria may be associated with poor outcomes in revision for infection and even suggested as a relative contraindication for single-stage exchange [16]. In this series, the infecting organism was a Gram-negative bacterium (*Klebsiella* and *Escherichia coli*) as well as MRSA in 25% of the patients, yet suitable antibiotics were available in both parenteral and oral forms. Hence, it is logical to consider the determining factor to be the availability of suitable ABs that can eradicate the bacteria when given in the adequate form and dose rather than solely the type of organism [6, 9, 13].

Availability of suitable antibiotics that can treat the infecting organism has been set as a prerequisite for single-stage revision [5, 25]. In this series, ABs were added to the cancellous graft in large doses (4 g/head). This high concentration of antibiotics within the hip space ensures eradication of the planktonic form and sessile clusters of the infecting bacteria as well as biofilm colonization [29].

Antibiotics were also given through parenteral route, commencing intraoperatively after debridement. Biofilm-active antimicrobial agents (rifampin and fluoroquinolones) were used for 8 weeks postoperatively.

One of the aims in revision hip arthroplasty is to restore the HCR as close to the anatomic position as possible. Hence, the size of the TMA would ideally correct any proximal migration of the cup. However, muscle tightness and soft tissue fibrosis may stand against complete correction of the hip mechanics. In this series it was possible to restore the HCR in 67% of the cases.

This study is not a randomized trial and includes a relatively small number of patients. However, it reports the results of employing TMA and AB-loaded bone graft for reconstruction of massive acetabular defects in single-stage revision for periprosthetic infection. This technique has recently been reported from a single unit, and it is important for the orthopedic community to watch the consistency and repeatability of the outcomes from different institutes. Longer-term follow-up and randomized trials will provide more conclusive evidence regarding choices for single or staged revision and techniques for reconstruction.
